# Supplementary material for: A scoping review of community knowledge in malaria prevention and control programmes
Source: PLoS One. 2025 Jul 21;20(7):e0328703. doi: 10.1371/journal.pone.0328703 (PMC12279157; doi:10.1371/journal.pone.0328703)
Supplement: Attachment — Submitted filename: R1,PLOS One,July2, 2025,Scoping Review Malaria Programmes.pdf [file pone.0328703.s004.pdf]

Reviewer Report

Manuscript #: PONE-D-25-20682R1

Title: A Scoping Review of Community Knowledge in Malaria Prevention and Control Programmes

Authors: Faizul Akmal Abdul Rahim1*, Mohd Amierul Fikri Mahmud1, Mohd Hatta Abdul Mutalip1, Norzawati Yoep1, Mohd Amiru Hariz Aminuddin1, Ahmad Mohiddin Mohd Ngesom1

Article type: Research Article

Review comments

Title

The authors have revised the title as suggested.

Abstract

The authors have revised as suggested.

Introduction

The authors have revised as suggested. However, the transition sentence beginning with "Moreover, it is increasingly …" to introduce socioeconomic status after discussing biomedical approaches remains abrupt. Instead of using "Moreover", it is better to change as "Understanding these non-biomedical influences, it is important to consider the broader social and economic factors that influence health behaviors and access to healthcare services."

Material and Methods

(1) Regarding with Q1, the authors have revised as suggested.
(2) For Q2, add a sentence specifying the method of agreement measurement or alternatively, if this was not done, they should acknowledge the limitation and it will consider more measures in future reviews.
(3) For Q3, add a sentence for rationale of why choosing only three databases such as their broad coverage of peer-reviewed biomedical literature and clarify whether additional sources were considered to ensure a comprehensive review.

Results

(1) Regarding with Q1, the authors added a summary Table 2.
(2) Regarding with Table (1), the authors have revised as suggested. However, the formatting still needs to be standardized, particularly the alignment and line spacing in the columns titled *"Community Knowledge of Malaria"* and *"The Role of Community Knowledge."*
(3) Regarding with Q3, the authors should clarify in the main text why not all studies assessed every aspect of community knowledge. For example, they could include a statement such as: *"Although our review included 63 studies, not all of them assessed every aspect of community knowledge. Specifically, knowledge of malaria transmission was evaluated in*

54 studies, symptoms in 40 studies, and prevention or control measures in 53 studies, depending on each study's focus and objectives."

(4) For Q4, the authors have revised the issue by adding it to the limitations section.

Discussion

(1) For Q1, the authors have revised as suggested. It is satisfactory.
(2) For Q2, the authors have revised as suggested.
(3) The authors have revised as suggested for Q3.
(4) The authors have added the discussion as suggested for Q4.
(5) For Q5, the authors have revised as suggested.
