# Supplementary material for: A scoping review of community knowledge in malaria prevention and control programmes
Source: PLoS One. 2025 Jul 21;20(7):e0328703. doi: 10.1371/journal.pone.0328703 (PMC12279157; doi:10.1371/journal.pone.0328703)
Supplement: Attachment — Submitted filename: Response to Reviewers.docx [file pone.0328703.s003.docx]

| Editor Comments | Author responses |
| --- | --- |
| - The study does not directly assess the impact of community knowledge on malaria outcomes. Instead, it maps and summarizes the existing literature on community knowledge regarding malaria prevention and control. Thus, "impact" should be omitted in the title. | - The revision has been made as instructed. The title was revised to “A Scoping Review of Community Knowledge in Malaria Prevention and Control Programmes” |
| - Additionally, the abstract and introduction suggest the review aims to assess the impact of community knowledge, but the methods only describe mapping knowledge, not evaluating outcomes or effectiveness. | - The revision has been made as instructed. **(refer to lines 14 and 64-66)** |
| - In the methods, clarify that the review did not extract or analyze data on the impact of knowledge, but rather on the extent and nature of community knowledge. Clarify it. | - The revision has been made as instructed. **(refer to lines 135-137)** |
| Reviewer #1 Comments | Author responses |
| - For the title, instead of “Malaria Programmes,” change to “Malaria Prevention” or “Malaria Prevention and Control Outcomes” or “Malaria Control and Elimination Efforts” or the term being more specific. | - The revision has been made as instructed. The title was revised to “A Scoping Review of Community Knowledge in Malaria Prevention and Control Programmes” |
| - Abstract: For methods, briefly describe the inclusion criteria (e.g., only peer-reviewed? specific populations?). | - The revision has been made as instructed. **(refer to lines 19-21).** - The review included studies across all population groups without limitation. |
| - Abstract: For results, briefly mention examples for “critical knowledge gaps”. | - The revision has been made as instructed. **(refer to lines 31-32).** |
| - Introduction: The introduction is well-structured and informative. However, transitions between some paragraphs are abrupt (e.g., from biomedical approaches to socioeconomic status). Use linking sentences to maintain a smooth flow. | - The revision has been made as instructed. **(refer to lines 58-59).** |
| - Material and Methods: Studies were included for original research published in English and exclusion of non-English studies introduces language bias, especially for malaria-endemic regions and excluding conference proceedings etc. may omit emerging evidence, which can be critical in rapidly evolving health issues like malaria. The community-based nature of the topic, reports from NGOs, ministries of health, or unpublished evaluations could provide valuable insights. | - We acknowledge that restricting the review to English-language, peer-reviewed articles may introduce language bias and exclude relevant grey literature such as conference abstracts, NGO reports, and unpublished evaluations, especially from malaria-endemic regions. This decision was made to ensure quality, manage translation resources, and maintain methodological rigor. We have added this as a limitation in the discussion and highlighted the need for future reviews to include non-English and grey literature to capture broader perspectives. |
| - Material and Methods: The manuscript mentioned that “a pilot sample of 20 articles was used to train the screeners, with a concordance rate exceeding 90% indicating their readiness to proceed.” Describe how agreement was measured (e.g., Cohen’s Kappa). | - Thank you for this insightful comment. Agreement during the pilot screening was assessed using percent agreement, calculated as the proportion of identical inclusion/exclusion decisions by the two reviewers on a sample of 20 articles. While we acknowledge that measures like Cohen’s Kappa account for chance agreement, percent agreement was deemed sufficient for training purposes to ensure consistent application of criteria. A threshold of over 90% was pre-set to indicate readiness. We have clarified this in the Study Selection section and will consider more robust measures in future reviews. |
| - Material and Methods: Only three databases (PubMed, Scopus and Web of Science) were searched. Justify why only these databases were used or expand the search to include additional sources to ensure a comprehensive literature review. | - We recognize the importance of comprehensive database coverage in systematic reviews. PubMed, Scopus, and Web of Science were selected for their broad, complementary indexing of biomedical, public health, and multidisciplinary literature. PubMed offers authoritative coverage of clinical and health science research, while Scopus and Web of Science extend to global health and social science domains relevant to our review. To balance comprehensiveness with feasibility and minimize duplication, we limited our search to these core databases. - Additionally, we manually screened the reference lists of included studies to identify any potentially relevant articles not captured in the initial search. We acknowledge that incorporating additional sources such as Embase or regional databases may further enhance coverage and will consider this in future reviews. |
| - Result: The results rely heavily on text and tables. Add summary tables or figures to present key findings more effectively for country-specific knowledge levels and practices. Include a summary of how knowledge or prevention behavior has changed over time. | - The revision has been made as instructed by adding a summary table. **(refer to Table 2)** |
| - Result: In Table (1), the year of the authors are randomly described. It is better to describe chronologically for clarity. | - The revision has been made as instructed. **(refer to Table 1)** |
| - Result: “Community knowledge of malaria transmission varied across regions, with 54 studies,” “Community knowledge of malaria symptoms was assessed in 40 studies,” “Community knowledge of malaria prevention and control measures was assessed in 53 studies,” regarding with these, why varied with 40, 53, 54 studies etc. instead of 62 studies? Describe the reasons for studies not include all studies areas. | - Although our review included 63 studies, not all assessed specific aspects of community knowledge. Specifically, knowledge of malaria transmission was evaluated in 54 studies, symptoms in 40 studies, and prevention or control measures in 53 studies. |
| - Result: “General awareness of antimalarial drugs was noted by 73.3% of respondents in Nigeria [47], 81.6% in Ethiopia [30], and 90% in Malaysia [8], reflecting varying levels of accessibility and health education initiatives.” In relation to these questions, were respondents aware of any species differences, such as Plasmodium falciparum and Plasmodium vivax, in terms of antimalarial drug treatment (Pf for AL and Pv for CQ)? | - Thank you for your insightful observation. In the included studies, respondents were not specifically asked about their awareness of Plasmodium species differences (*P. falciparum* vs *P. vivax*) to antimalarial drug treatment. The majority of the surveys assessed general community knowledge regarding malaria symptoms, transmission, and commonly used treatment drugs (e.g., artemether-lumefantrine, chloroquine), without differentiating by parasite species. - The limitation was included in the study limitations. **(refer to lines 431-437)** |
| - Discussion: Some statements, like “Community knowledge levels strongly influenced the ownership and use of mosquito nets...” assume broad regional trends without specifying the variability within countries or among different community types (urban vs. rural, indigenous vs. non-indigenous, etc.). If possible, quantify or categorize heterogeneity (e.g., rural vs urban settings, year ranges) the findings and discuss or acknowledge it. | - Thank you for this valuable comment. We acknowledge that our original discussion generalized regional findings without sufficiently addressing the intra-country and community-level heterogeneity in knowledge and prevention practices. A closer examination of the included studies revealed that only a limited number reported differences by community type (e.g., urban versus rural) and sociodemographic context (e.g., indigenous status, education levels). However, such details were not consistently available across all studies, limiting our ability to quantitatively synthesize these subgroups. - We provide a discussion on this variability in the discussion **(refer to lines 364-375)** and the study limitations (**refer to lines 423-426)** |
| - Discussion: The discussion does not sufficiently link the findings to practical applications or broader policy implications. Compare the findings more explicitly to prior reviews or studies, particularly those reporting conflicting or supporting evidence, and explain how this review contributes uniquely to the field. Discuss more targeted suggestions, such as: incorporating malaria education into school curricula, utilizing mobile health tools to reach remote areas and training local community health workers to sustain education efforts. | - Thank you for your insightful comment. We appreciate the suggestion to enhance the discussion by drawing clearer links between our findings, prior research, and policy implications. **(refer to lines 399-409)** |
| - Discussion: The discussion does not mention how gender, age, or educational level affect community knowledge or malaria prevention practices. These demographic factors are known to significantly influence health behaviour and access to information, discuss it, if data are available. | - The revision has been made as instructed. **(refer to lines 376-383).** |
| - Discussion: The discussion focuses mainly on community knowledge, but less so on the health system’s role for behaviour change. Although community education is vital, it must be supported by a strong health system including reliable supply chains, well-trained personnel, and accessible facilities to ensure that increased awareness leads to translate knowledge into effective action. Add those in discussion. | - Thank you for this important and insightful comment. We agree that community knowledge alone is not sufficient to drive sustainable behaviour change without the support of a robust and responsive health system. In response, we have revised the discussion to acknowledge the critical interplay between community-level awareness and system-level readiness. **(refer to lines 392-398)** |
| - Discussion: There's little discussion of global comparisons that how the findings from Africa and Asia compare to or inform malaria responses in other parts of the world. | - Thank you for this thoughtful observation. We acknowledge that our original discussion focused primarily on findings from Africa and Asia, which represent the majority of included studies due to the high malaria burden in these regions. However, we agree that providing a broader global perspective can enhance the utility and relevance of the findings. - We have now revised the discussion to include a comparative reflection on how insights from Africa and Asia can inform malaria strategies in other endemic or re-emerging regions, such as parts of South America and Oceania. **(refer to lines 322-329)** |
| Reviewer #2 Comments | Author responses |
| - As 69.4% of the included studies are from African countries, the findings may disproportionately reflect the context of that region. This geographic concentration may limit the generalizability of the conclusions and may not fully represent global patterns of community knowledge on malaria. | - Thank you for this important observation. We fully agree that the geographic distribution of included studies, predominantly from Africa, reflects both the disproportionate malaria burden in that region and the research focus of the global health community. While this concentration provides rich insight into diverse African contexts, we acknowledge that it may limit the global generalizability of the findings. **(refer to lines 322-329)** |
| - Method: The search strategy should not be restricted to terms such as ‘community knowledge’ or ‘population knowledge,’ as this may exclude relevant studies on malaria-related knowledge conducted at the community level that do not explicitly use these terms in their titles or keywords. For example, some studies are conducted in malaria-endemic villages but may not describe the setting as ‘community-based.’ Including such studies could enhance the comprehensiveness and representativeness of the review findings. | - Although our search strategy focused on terms such as 'community knowledge' and 'population knowledge,' we also included studies that described knowledge and practices at the village or household level, even if the term 'community-based' was not explicitly used. Nonetheless, we acknowledge that some relevant studies may have been missed due to terminology differences. Future reviews may benefit from incorporating broader terms such as 'household survey,' 'village-level knowledge,' and 'malaria awareness' to improve sensitivity and capture studies with community-relevant data that use alternative descriptors. - We included a study in the malaria endemic area of Myanmar as the reviewer’s suggestion. **(refer to Table 2, number 41)** |
| - Result: Please verify the data presented in Figure 1. The total number of records identified from the three databases (538 + 213 + 21) should be 772, and any discrepancy should be clarified. | - The revision corrects the number of studies identified from Web of Science to 91, instead of the previously stated 21. |
| - Result: The demographic characteristics of the study participants, particularly socioeconomic status, should be clearly reported. As noted in the introduction, community knowledge is linked to socioeconomic factors; thus, providing this information would strengthen the interpretation and contextualization of the findings. | - Thank you for this valuable observation. We agree that socioeconomic status (SES) is a key determinant of community knowledge and behaviour related to malaria, and reporting this information would enhance the contextual interpretation of our findings. - Upon review of the included studies, we found that while many reported basic demographics such as age, gender, and education level, fewer provided detailed data on income, occupation, or composite SES indices. Where such data were available, they were often described qualitatively or inconsistently across studies, which limited our ability to conduct a comparative synthesis. - The revision has been made as instructed. **(refer to lines 384-392).** |
| - Discussion: Malaria treatment practices vary by Plasmodium species. The authors should discuss community knowledge regarding species-specific treatments, if applicable, to provide a more understanding in both result and discussion sections. | - Thank you for your insightful observation. In the included studies, respondents were not specifically asked about their awareness of Plasmodium species differences (*P. falciparum* vs *P. vivax*) to antimalarial drug treatment. The majority of the surveys assessed general community knowledge regarding malaria symptoms, transmission, and commonly used treatment drugs (e.g., artemether-lumefantrine, chloroquine), without differentiating by parasite species. - The limitation was included in the study limitations. **(refer to lines 431-437)** |
| - Discussion: Line 349: The term ‘outdated treatments’ may be considered imprecise in a scientific context. It is recommended to replace it with ‘no longer recommended as first-line treatment due to widespread resistance in some countries,’ which more accurately reflects current clinical guidelines. | - The revision has been made as instructed. **(refer to lines 340-341).** |
| - Discussion: Given that one of the study’s objectives is to inform culturally appropriate approaches in global malaria control efforts, the authors should expand the discussion to include culturally tailored malaria interventions, supported by relevant reference studies. | - Thank you for this suggestion. We agree that culturally tailored interventions are essential for the success and sustainability of malaria control efforts, particularly in diverse sociocultural settings. In response, we have expanded the discussion to include examples of effective culturally sensitive approaches and referenced relevant studies that demonstrate their impact. **(refer to lines 357-363)** |
