# Supplementary material for: A scoping review of community knowledge in malaria prevention and control programmes
Source: PLoS One. 2025 Jul 21;20(7):e0328703. doi: 10.1371/journal.pone.0328703 (PMC12279157; doi:10.1371/journal.pone.0328703)
Supplement: Attachment — Submitted filename: Response_to_Reviewers_auresp_2.docx [file pone.0328703.s005.docx]

| Journal Requirement | Author responses |
| --- | --- |
| - Please review your reference list to ensure that it is complete and correct. If you have cited papers that have been retracted, please include the rationale for doing so in the manuscript text, or remove these references and replace them with relevant current references. Any changes to the reference list should be mentioned in the rebuttal letter that accompanies your revised manuscript. If you need to cite a retracted article, indicate the article’s retracted status in the References list and also include a citation and full reference for the retraction notice. | - All 88 references cited in the manuscript have been thoroughly reviewed using Retraction Watch, PubMed, and publishers’ official databases. No retractions, expressions of concern, or formal corrections were identified. All sources remain valid and appropriate for citation. - Should any retracted articles have been identified on your end, I would greatly appreciate it if you could kindly indicate them. This would allow me to revise the manuscript accordingly by removing or replacing the affected references. Thank you. |
| Reviewer #1 Comments | Author responses |
| - Introduction: The authors have revised as suggested. However, the transition sentence beginning with “Moreover, it is increasingly …” to introduce socioeconomic status after discussing biomedical approaches remains abrupt. Instead of using “Moreover”, it is better to change as “Understanding these non-biomedical influences, it is important to consider the broader social and economic factors that influence health behaviors and access to healthcare services.” | - The revision has been made as instructed. **(refer to lines 58-59)** |
| - Material and Methods: For Q2, add a sentence specifying the method of agreement measurement or alternatively, if this was not done, they should acknowledge the limitation and it will consider more measures in future reviews. | - The revision has been made as instructed in the study limitations. **(refer to lines 467-473)** |
| - Material and Methods: For Q3, add a sentence for rationale of why choosing only three databases such as their broad coverage of peer-reviewed biomedical literature and clarify whether additional sources were considered to ensure a comprehensive review. | - The revision has been made as instructed. **(refer to lines 104-114).** |
| - Results: Regarding with Table (1), the authors have revised as suggested. However, the formatting still needs to be standardized, particularly the alignment and line spacing in the columns titled “Community Knowledge of Malaria” and “The Role of Community Knowledge.” | - The revision has been made as instructed. **(refer to Table 1)** |
| - Results: Regarding with Q3, the authors should clarify in the main text why not all studies assessed every aspect of community knowledge. For example, they could include a statement such as: “Although our review included 63 studies, not all of them assessed every aspect of community knowledge. Specifically, knowledge of malaria transmission was evaluated in 54 studies, symptoms in 40 studies, and prevention or control measures in 53 studies, depending on each study’s focus and objectives.” | - The revision has been made as instructed. **(refer to lines 156-160)** |
