# Supplementary material for: A scoping review of community knowledge in malaria prevention and control programmes
Source: PLoS One. 2025 Jul 21;20(7):e0328703. doi: 10.1371/journal.pone.0328703 (PMC12279157; doi:10.1371/journal.pone.0328703)
Supplement: Attachment — Submitted filename: Reviewer comment .pdf [file pone.0328703.s002.pdf]

Dear Editor and authors,

Thank you very much for the opportunity to review this manuscript. The manuscript presents a systematic review exploring the impact of community knowledge on malaria control across 27 countries.

The below are some comments to improve the manuscript.

General Comment

As 69.4% of the included studies are from African countries, the findings may disproportionately reflect the context of that region. This geographic concentration may limit the generalizability of the conclusions and may not fully represent global patterns of community knowledge on malaria.

Method

The search strategy should not be restricted to terms such as ‘community knowledge’ or ‘population knowledge,’ as this may exclude relevant studies on malaria-related knowledge conducted at the community level that do not explicitly use these terms in their titles or keywords. For example, some studies are conducted in malaria-endemic villages but may not describe the setting as ‘community-based.’ Including such studies could enhance the comprehensiveness and representativeness of the review findings.

Examples:

https://www.scopus.com/record/display.uri?eid=2-s2.0-85077859331&origin=resultslist&sort=plf-f&src=s&sot=b&sdt=b&s=TITLE-ABS-KEY%28Knowledge%2C+attitude+and+practice+levels+regarding+malaria+among+people+living+in+the+malaria+endemic+area+of+Myanmar%29&sessionSearchId=6b578ac9c29c120d46905b72f71c98e8

https://www.scopus.com/record/display.uri?eid=2-s2.0-85147000334&origin=resultslist&sort=plf-f&src=s&sot=b&sdt=b&s=TITLE-ABS-KEY%28malaria+and+knowledge+and+Myanmar%29&sessionSearchId=e031fe178e5de498186de3c3faa2b040&relpos=8

Result

- Please verify the data presented in Figure 1. The total number of records identified from the three databases (538 + 213 + 21) should be 772, and any discrepancy should be clarified.
- The demographic characteristics of the study participants, particularly socioeconomic status, should be clearly reported. As noted in the introduction, community knowledge is linked to socioeconomic factors; thus, providing this information would strengthen the interpretation and contextualization of the findings.

Discussion

- Malaria treatment practices vary by *Plasmodium* species. The authors should discuss community knowledge regarding species-specific treatments, if applicable, to provide a more understanding in both result and discussion sections.
- Line 349: The term ‘outdated treatments’ may be considered imprecise in a scientific context. It is recommended to replace it with ‘no longer

recommended as first-line treatment due to widespread resistance in some countries,' which more accurately reflects current clinical guidelines.

- Given that one of the study's objectives is to inform culturally appropriate approaches in global malaria control efforts, the authors should expand the discussion to include culturally tailored malaria interventions, supported by relevant reference studies.
